# Hereditary angioedema care across selected health systems in the Balkan Peninsula area: policy gaps, practice variation, and actionable recommendations

**DOI:** 10.3389/falgy.2026.1871835

**Published:** 2026-07-20

**Authors:** George N. Konstantinou, Sladjana Andrejevic, Natasha Angjeleska, Elif Karakoc-Aydiner, Noémi Anna Bara, Marko Barešić, Krasimira Baynova, Cathrine Chliva, Ljerka Čulav, Nihal Mete Gökmen, Mensuda Hasanhodžić, Mehmet Hoxha, Gül Karakaya, Eris Mesonjesi, Radovan Mijanovic, Maria Staevska, Efthalia Stefanaki, Fotis Psarros, Matija Rijavec, Paraskevi Xepapadaki, Mihaela Zidarn, Anastasios E. Germenis, Anna Valerieva

**Affiliations:** 1 Department of Allergy and Clinical Immunology, 424 General Military Training Hospital, Thessaloniki, Greece; 2 Clinic of Allergy and Immunology, University Clinical Center of Serbia, Belgrade, Serbia; 3 School of Medicine, University of Belgrade, Belgrade, Serbia; 4 Department of Psychology, School of Political Sciences, University American College Skopje, Skopje, North Macedonia; 5 HAE International, Fairfax City, VA, United States; 6 Department of Pediatrics, Division of Allergy and Immunology, Istanbul Jeffrey Modell Diagnostic and Research Center for Primary Immunodeficiencies, The Isil Berat Barlan Center for Translational Medicine, Immune Deficiency Research and Application Center, Istanbul, Türkiye; 7 Centrul Clinic MediQuest, Romanian Angioedema Center of Reference and Excellence, Sângeorgiu de Mureş, Romania; 8 Division of Clinical Immunology and Rheumatology, Department of Medicine, School of Medicine, University of Zagreb, University Hospital Center Zagreb, Zagreb, Croatia; 9 Spanish National Center for Hereditary Angioedema, Allergy Unit, Virgen del Rocío University Hospital, Seville, Spain; 10 Allergy Unit “D. Kalogeromitros”, 2nd Department of Dermatology and Venereology, Medical School, University General Hospital Attikon, Haidari, Greece; 11 General Hospital Šibenik, Šibenik, Croatia; 12 Faculty of Medicine, Department of Internal Medicine, Division of Allergy and Immunology, Ege University, Izmir, Türkiye; 13 Center for Rare Diseases, University Clinical Center Tuzla, Tuzla, Bosnia and Herzegovina; 14 Department of Allergology and Clinical Immunology, University Hospital Center Mother Teresa, Medical University Tirana, Tirana, Albania; 15 Faculty of Medicine, Department of Chest Diseases, Division of Allergy and Immunology, Hacettepe University, Ankara, Türkiye; 16 Department of Allergology, University Hospital Alexandrovska, Medical University of Sofia, Sofia, Bulgaria; 17 Private Allergy Practice, Ioannina, Greece; 18 Allergy Clinic, Athens Naval Hospital, Athens, Greece; 19 University Clinic of Respiratory and Allergic Diseases Golnik, Golnik, Slovenia; 20 Biotechnical Faculty, University of Ljubljana, Ljubljana, Slovenia; 21 Faculty of Medicine, University of Maribor, Maribor, Slovenia; 22 Allergy Department, 2nd Pediatric Clinic, National and Kapodistrian University of Athens, Athens, Greece; 23 Faculty of Medicine, University of Ljubljana, Ljubljana, Slovenia; 24 Department of Immunology and Histocompatibility, School of Medicine, University of Thessaly, Larissa, Greece

**Keywords:** hereditary angioedema, Balkan Peninsula, health services accessibility, diagnostic delay, reimbursement, on-demand treatment, long-term prophylaxis, self-administration

## Abstract

Hereditary angioedema is a rare but potentially life-threatening disorder in which outcomes depend not only on correct diagnosis and effective medicines, but also on how health systems organize referral, laboratory confirmation, emergency pathways, reimbursement, home treatment, and long-term follow-up. Selected health systems in the Balkan Peninsula area provide a particularly informative setting for health-system comparison because neighboring countries with active hereditary angioedema expertise differ substantially in rare-disease governance, registry maturity, diagnostic infrastructure, treatment coverage, and patient-organization capacity. This Policy and Practice Review synthesizes international guidance, published regional literature, comparative country information from the Balkan Experts in Angioedema: Consensus and Ongoing Navigation (BEACON) initiative, and advocacy-informed implementation insights to assess current care delivery in Albania, Bosnia and Herzegovina, Bulgaria, Croatia, Greece, Romania, Serbia, Slovenia, and Türkiye. Across the region, the most consistent problems are prolonged diagnostic delay, unequal access to complement and genetic testing, approved-but-not-reimbursed modern therapies, hospital-only access to rescue medication, uneven use of home treatment and self-administration, incomplete emergency preparedness, and variable registry and advocacy infrastructure. Countries with stronger alignment between policy frameworks, specialist centers, registries, reimbursement pathways, and patient organizations appear better positioned to deliver guideline-concordant care, whereas fragmentation at any point in the care pathway reduces the practical value of therapeutic advances. The review argues that the main barriers to equitable hereditary angioedema care across the included health systems are now predominantly regulatory, financing, organizational, and educational rather than scientific. We therefore propose actionable recommendations for ministries and payers, specialist centers and professional societies, emergency-care systems, registry stakeholders, and patient organizations, with the goal of converting regional heterogeneity into a structured quality-improvement agenda.

## GRAPHICAL ABSTRACT

**Figure:**
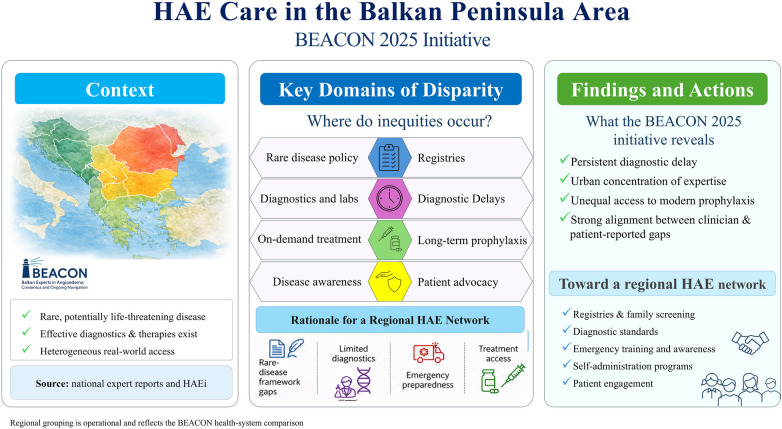

## 1 Introduction

Hereditary angioedema (HAE) is a bradykinin-mediated disease characterized by recurrent, self-limiting episodes of subcutaneous and submucosal swelling that can affect the skin, gastrointestinal tract, and upper airway. The primary hereditary types are HAE caused by C1 inhibitor deficiency or dysfunction (HAE-C1INH) and HAE with normal C1 inhibitor (HAE-nC1INH). Acquired C1 inhibitor deficiency, a distinct form of bradykinin-mediated angioedema, can resemble HAE-C1INH and is important in differential diagnosis and certain registry classifications (1–4). Although HAE is rare, the consequences of delayed diagnosis or inadequate treatment are clinically disproportionate: abdominal attacks can lead to repeated emergency consultations and unnecessary surgery, while laryngeal attacks remain potentially fatal.

Modern HAE management is no longer defined only by the availability of a specific medicine. Instead, care should be understood as a cascade that begins with clinical suspicion and continues through biochemical confirmation, subtype classification, family screening, education, emergency planning, access to on-demand treatment (ODT), and individualized evaluation for long-term prophylaxis (LTP). The 2021 revision of the international WAO/EAACI guideline emphasizes early treatment of attacks, avoidance of ineffective anti-allergic therapy for bradykinin-mediated swelling, and consideration of self-administration and prophylaxis according to disease activity and patient need (2). Consensus work on angioedema terminology and classification has further clarified the importance of distinguishing disease mechanisms if care pathways are to be clinically coherent (4).

The burden of HAE extends beyond attack frequency. Qualitative and quantitative work has shown that the disease affects schooling, work participation, family life, mental well-being, and health-related quality of life (5, 6). Communication failures, disease misunderstanding, and inequities in healthcare resources can further amplify that burden for both patients and clinicians (7, 8). In addition, HAE remains vulnerable to system-level failure. A patient may reach an expert clinic late because primary or emergency care does not recognize bradykinin-mediated swelling; a patient with a correct diagnosis may still lack timely access to rescue therapy because stock is centralized, reimbursement is incomplete, or home treatment has not been authorized. Patient advocacy and policy discussions in HAE, therefore, increasingly focus on how scientific progress translates into real-world access across countries (9, 10).

Selected health systems in the Balkan Peninsula area are particularly relevant for this discussion. The countries included in this health-system comparison are geographically proximate, medically interconnected, and active contributors to HAE scholarship, yet they differ in population size, rare-disease planning, procurement systems, health-financing models, referral geography, and registry infrastructure. Published regional work includes family and cohort studies from Croatia, Greece, Serbia, Slovenia, Türkiye, and North Macedonia, as well as national guidance and registry reports, demonstrating that the region contributes to the scientific literature rather than merely consuming it (11–22). More specifically, a reproducible PubMed snapshot, provided in Supplementary Data Sheet 1, identified 186 of 2,691 PubMed-indexed HAE publications (7%) from 1 January 2000 to 12 September 2025 with at least one Balkan Peninsula area co-author; corresponding figures were 17 of 162 clinical-trial or randomized-trial publications (11%) and 5 of 52 title-filtered guideline or consensus publications (10%). These pragmatic data show that the region is an active contributor to HAE knowledge, while the practical availability of modern HAE diagnostics and therapies remains uneven.

The BEACON (Balkan Experts in Angioedema: Consensus and Ongoing Navigation) initiative was created to support regional exchange on these implementation problems. Its value lies less in generating novel efficacy data than in allowing clinicians and patient advocates to compare how policy decisions, reimbursement rules, and service organization shape patient care. The central question is not whether effective diagnostics and treatments for HAE exist; that question is largely settled. The more urgent question is whether the included health systems enable a patient with HAE to receive guideline-concordant, rapid, and safe care in everyday practice.

This review therefore aims to assess the policy and practice landscape of HAE care across Albania, Bosnia and Herzegovina, Bulgaria, Croatia, Greece, Romania, Serbia, Slovenia, and Türkiye; identify the implementation bottlenecks that most strongly shape inequity; and translate those observations into actionable recommendations for ministries of health, payers, specialist centers, professional societies, emergency systems, registry stakeholders, and patient organizations.

## 2 Methodological approach and evidence sources

This article synthesizes four evidence streams: i) international guidance and consensus documents relevant to HAE diagnosis, classification, and management (1, 2, 4); ii) published regional literature describing national cohorts, registries, diagnostic experiences, molecular findings, and country-level policy or practice developments (10–22); iii) country reports and working materials prepared for the BEACON 2025 regional exchange by participating clinicians; and iv) advocacy-informed implementation insights discussed with patient representatives during the same exchange. No identifiable patient-level data were collected or analyzed for this review.

The policy-mapping exercise focused on domains that directly influence equitable care delivery: rare-disease governance, existence and organization of specialist centers, known and expected patient numbers, diagnostic delay, availability of C4, C1 inhibitor antigen, C1 inhibitor function, C1q, anti-C1 inhibitor antibodies, and genetic testing, access to ODT and LTP, home treatment and self-administration, emergency preparedness, registry infrastructure, patient organizations, and reported gaps in innovation or clinical trial participation. Countries were included when sufficiently detailed and internally coherent BEACON health-system information was available for synthesis. Inclusion should therefore be understood as operational and evidence-based rather than as a formal geographic or geopolitical classification. North Macedonia, Montenegro, and Kosovo are not part of the comparative country table because equivalent BEACON mapping material was not available at the time of drafting, although relevant published North Macedonian data are acknowledged in the discussion (22).

A central methodological distinction was made between regulatory approval, practical availability, reimbursement, and home use. A medicine may be licensed nationally yet not publicly reimbursed, reimbursed but available only in a tertiary hospital, or stocked in a referral center without a reliable pathway for rapid community access. Similarly, a test may be technically available but functionally inaccessible because of cost, transport logistics, private-only provision, or the need to send samples abroad. This distinction is crucial in HAE because apparent access on paper may not translate into timely treatment during abdominal or laryngeal attacks. The same logic applies to prophylaxis. Reimbursement status alone does not show whether patients can actually start and continue treatment. In this context, initiation criteria include attack-severity thresholds, specialist authorization, age limits, renewal requirements, and documentation rules. Together with administrative barriers, geography, and follow-up arrangements, these factors determine whether reimbursement translates into meaningful patient benefit.

Because the review is concerned with system organization and policy implementation, the goal was not to rank countries or to produce pooled epidemiologic estimates. Reported patient numbers should therefore be interpreted as country-level estimates or known case counts within each national context, not as standardized prevalence studies. Nevertheless, comparative analysis remains informative because recurring bottlenecks are visible across multiple settings. Where similar problems appear in countries with different sizes and health-financing structures, those problems are more likely to represent structural features of HAE care than isolated local anomalies.

The policy-review format also shaped the treatment of non-traditional sources. Published academic literature anchors the clinical and conceptual framework of the article, while BEACON materials were used to describe current service organization and implementation realities at a system level. HAEi State of Management material is cited as contextual advocacy literature (9), but the review does not treat advocacy material as a substitute for peer-reviewed evidence. Instead, it uses these materials to illuminate where formal policy or reimbursement arrangements fail to align with patients' lived needs.

To improve transparency, Supplementary Data Sheet 1 reproduces the PubMed search strategy used for the bibliometric snapshot mentioned in the introduction, and Supplementary Data Sheet 2 documents the operational labels applied during policy mapping. These supplementary files are not intended to convert the manuscript into a systematic review; rather, they allow readers to understand how the authors moved from heterogeneous country-level information to a comparable analytic framework. This is particularly important in policy reviews, where conceptual ambiguity around terms such as “available”, “covered”, or “center” can create false equivalence across systems.

**Table 1:** Country-level policy and practice checklist for hereditary angioedema infrastructure, diagnostics, treatment access, and innovation status in selected health systems in the Balkan Peninsula area.

Country	Burden/specialist network	Policy checklist	Diagnostics	Access checklist	Trials/novel status/key gap
Albania	Pop. 2.75M 45 diagnosed (≈55 expected) mean age 40.2 y Tirana tertiary center.	Rare disease plan: no dedicated plan. HAE registry: no. Patient support: Rare Disease Association + HAEi contact.	C4 yes; C1INH antigen/function limited and not publicly reimbursed; genetics private/abroad. Delay: 10 y (0.5–26).	ODT: rhC1INH + FFP, mainly tertiary. Home ODT: not established. LTP: lanadelumab for 2 patients; danazol still used.	Trials: none reported. Novel status: very limited reimbursed access to modern prophylaxis. Key gap: reimbursed diagnostics and therapy access.
Bosnia and Herzegovina	Pop. 3.11M 13 in HAE registry (≥44 expected) decentralized care.	Rare disease plan: entity-level only. HAE registry: yes. Patient support: HAEi contact + HAE association pending final registration.	C4/C1INH/C1q covered in several facilities; family screening covered; genetics abroad, mostly reimbursed/project-funded. Delay: 14.9 y (1–47).	Approved: rhC1INH, icatibant, lanadelumab, berotralstat. Available/reimbursed: only rhC1INH reported available; drug costs not reimbursed; FFP nationwide.	Trials: none reported. Novel status: modern drugs approved but not reimbursed/available. Key gap: reimbursement, procurement and emergency protocols.
Bulgaria	Pop. 6.67M 150 diagnosed from 44 families (≈185 expected) Sofia expert/registry center.	Rare disease plan: expired (2009–2013). HAE registry: yes, since 1972. Patient support: yes.	C4/C1INH/C1q in specialist labs but not publicly reimbursed; C1INH function/genetics may require collaboration/abroad. Delay: 10.2–13.9 y.	ODT: pd/rhC1INH + icatibant reimbursed. LTP: lanadelumab/berotralstat reimbursed for eligible patients; special routes for younger children.	Trials/novel: donidalorsen and sebetralstat trials reported. Key gap: reimbursement rule covers either ODT or LTP per patient.
Croatia	Pop. 3.82M approximately 120 diagnosed nationally (BEACON materials detail 98 adults and 18 minors; ≈150 expected) 7 HAE centers.	Rare disease plan: new program under development; National Commission established 2023. HAE registry/network: national specialist network. Patient support: HAE Hrvatska.	C1INH antigen/function at 7 centers; C1q by referral/special arrangement; SERPING1 at Zagreb; broader genetics via Slovenia. Delay: median 13 y (5–43).	ODT: icatibant + pd/rhC1INH, hospital or home. LTP: androgens, tranexamic acid, lanadelumab and berotralstat.	Trials: clinical trials reported. Key gap: physician education, expanded diagnostics and improved prophylaxis access.
Greece	Pop. 9.90M 185 diagnosed in 55 families (200–300 estimated) 2 ACARE centers, 1 National Center of Excellence, pediatric/adolescent clinic, 24/7 helpline.	Rare disease/HAE framework: established. HAE registry: national initiative since 2009. Patient support: national HAE association.	Complement diagnostics and genetic testing available; national free genetic testing initiative for patients/relatives. Delay: ≈13 y.	ODT: IV C1INH and self-administered icatibant widely available. LTP: lanadelumab, berotralstat, SC C1INH and garadacimab reimbursed.	Trials/novel: sebetralstat trial reported. Key gap: island/rural access, ED/ENT awareness and psychosocial support.
Romania	Pop. 18.80M 176 HAE type I/II + 1 HAE-nC1INH (190–350 estimated) national HAE/ACARE center.	Rare disease plan: Health Strategy 2024–2030 with funding. HAE registry: since 2006. Patient support: two HAEi-affiliated groups.	C4 reimbursed; C1INH antigen/function free via sponsored project; C1q private; genetics by collaboration or out-of-pocket. Delay: 15.6 y in 84 patients.	ODT: pdC1INH, icatibant and FFP. Coverage: all HAE treatments 100% reimbursed, including uninsured patients. Home: specific IV/SC treatments approved.	LTP: IV/SC pdC1INH, lanadelumab, berotralstat; rare androgen use via procurement abroad. Trials: clinical trials reported. Key gap: genetic testing, access to newer therapies, and ED education.
Serbia	Pop. 6.64M 118 HAE-C1INH treatment approved for 96 patients multiple major-city sites.	Coverage: mandatory public insurance. Rare disease registry: national register. Patient support: HAE Srbija.	C3/C4 widely available; C1INH antigen in multiple centers; C1q/function in key institutions; anti-C1INH antibodies unavailable; funded NGS via IMGGI. Delay: 12.5 y.	ODT: pd/rhC1INH and icatibant available. LTP: lanadelumab, berotralstat; first SC pdC1INH LTP patient in Feb 2026.	Trials: clinical trials reported. Key gap: self-administration training, local stock control and supply continuity.
Slovenia	Pop. 2.11M 28 HAE-C1INH + 2 HAE-FXII under follow-up (≈50 expected) national HAE center.	Rare disease plan: yes. Registry: integrated registry-based approach. Patient support: HAE Slovenia linked to HAEi.	Complement and advanced genetics covered when indicated; specialized molecular testing domestic or collaborative. Delay: median 13 y; up to 40 y historically.	ODT: IV pd/rhC1INH and icatibant. Home: covered. LTP: IV C1INH, lanadelumab, berotralstat for selected patients; danazol only in few long-standing cases.	Trials: none reported. Key gap: family screening and HAE-nC1INH awareness.
Türkiye	Pop. 87.93M 658 in HAE/AAE-TR combined registry across 55 centers/24 cities > 7,500 undiagnosed estimated.	Rare disease plan: 2023–2027. Ministry rare diseases department: yes. Registry: HAE/AAE-TR. Patient support: HAÖDER.	Complement/genetics fully available and reimbursed at major referral centers; outsourcing elsewhere; C1q/anti-C1INH antibodies only at Ege. Delay: 15.4 y (median 12; max 76).	ODT: pdC1INH hospital-only; icatibant home use reimbursed; rhC1INH not approved. LTP: danazol/tranexamic acid covered; 34% use danazol; lanadelumab/berotralstat approved, not reimbursed.	Trials/novel: donidalorsen trial reported; named-patient import for unregistered drugs. Key gap: modern prophylaxis reimbursement and decentralized advanced diagnostics.

Patient numbers and access status are time-stamped to the BEACON 2025 source material unless a later date is explicitly stated. Expected patient counts are approximate because estimation methods differ between countries.

Population source: Worldometer country population estimates (2026), based on latest United Nations Population Division estimates; accessed 19 April 2026.

Clinical-trial status indicates whether an HAE-related clinical trial or named investigational therapy was reported in the BEACON working table; it does not imply nationwide access.

Türkiye: *n* = 658 refers to the combined HAE/AAE-TR registry, which includes HAE-C1INH, HAE-nC1INH, and acquired angioedema.

AAE, acquired angioedema; ACARE, Angioedema Centers of Reference and Excellence; BEACON, Balkan Experts in Angioedema: Consensus and Ongoing Navigation; C1INH, C1 inhibitor; C1q, complement C1q; C3, complement C3; C4, complement C4; ED, emergency department; FFP, fresh frozen plasma; HAE, hereditary angioedema; HAE-C1INH, HAE due to C1 inhibitor deficiency/dysfunction; HAE-FXII, HAE with F12 mutation; HAEi, HAE International; HAE-nC1INH, HAE with normal C1 inhibitor; IMGGI, Institute of Molecular Genetics and Genetic Engineering; IV, intravenous; LTP, long-term prophylaxis; NGS, next-generation sequencing; ODT, on-demand treatment; pd/rhC1INH, plasma-derived/recombinant C1 inhibitor; SC, subcutaneous.

## 3 Regional country-by-country overview

Before turning to the cross-country thematic analysis, it is useful to present a concise country-by-country overview of the regional landscape. These summaries are intended to preserve implementation-level detail that may be diluted in higher-level synthesis and to provide the necessary context for Table 1 and for the comparative policy and practice analysis that follows.

### 3.1 Albania

Albania reports no dedicated national rare-disease plan or HAE registry; rare diseases are only partly represented in broader health strategies. Care is concentrated at University Hospital Center “Mother Teresa”, Tirana, with support from the Center for Rare Diseases. Complement component 4 (C4) testing is available; C1 inhibitor antigen and function testing are limited and not publicly reimbursed; genetic testing is private or sent abroad. The presentation reported 45 diagnosed HAE patients (18 male, 27 female), mean age 40.2 years, and an average diagnostic delay of 10 years (range 0.5–26). ODT includes recombinant C1 inhibitor and fresh frozen plasma (FFP), mainly through the tertiary center. Lanadelumab became available for two patients in April 2025, whereas danazol remains in use. Priorities are publicly available and reimbursed diagnostics, broader access to modern therapies, registry development, physician education, and support for the Rare Disease Association of Albania and the HAEi-recognized national contact.

The Albanian situation shows how centralization can preserve expertise while still leaving patients exposed to delay. If diagnostic confirmation and treatment initiation depend mainly on one tertiary pathway, patients outside Tirana may experience longer routes to recognition, and emergency clinicians may be less familiar with HAE. A practical first step would be a national diagnostic algorithm linked to sample referral and reimbursement, combined with emergency-service education and family screening once an index case is diagnosed.

### 3.2 Bosnia and Herzegovina

Bosnia and Herzegovina illustrates the complexity of a decentralized system. No single national rare-disease plan is reported, but entity-level programs, solidarity funds, and ongoing legislative or registry efforts exist. A country-wide HAE registry has been established; BEACON material reported 13 diagnosed patients versus at least 44 expected. C1 inhibitor antigen and function, C4, and complement C1q are available in several laboratories or healthcare facilities and are covered or reimbursed; family screening is also covered. Genetic testing is performed abroad, with costs mostly reimbursed subsequently or project-funded. Diagnostic delay averages 14.9 years (range 1–47), mainly in older patients, and some older patients died before HAE confirmation. Four HAE medicines are agency-approved—recombinant C1 inhibitor, icatibant, lanadelumab, and berotralstat—whereas androgens and tranexamic acid are not approved for HAE. Only recombinant C1 inhibitor is reported as practically available, and HAE drug costs are not reimbursed, so patients may seek treatment abroad. FFP is available nationally, but undiagnosed attacks may still be treated incorrectly as allergic angioedema. Rare-disease associations are active, an HAEi-recognized HAE contact exists, and the Federation HAE association is in final legal registration.

For Bosnia and Herzegovina, the main implementation issue is coordination across administrative units. Diagnostic confirmation may occur, but it does not reliably trigger access to disease-specific treatment when reimbursement, procurement, and practical availability are not aligned. Regional guidance and emergency protocols could help standardize minimum care while reimbursement solutions are negotiated.

### 3.3 Bulgaria

Bulgaria adopted an early national rare-disease plan for 2009–2013, but no successor plan has been formally adopted. In contrast, HAE-specific expertise is long-standing: a national registry has been maintained at the Clinic of Allergology of Alexandrovska University Hospital since 1972, and Bulgarian clinicians have contributed to regional discussions on treatment availability (10). Current reports from the Bulgarian HAE/angioedema clinical dataset include 150 patients from 44 unrelated families, with hereditary categories including HAE-C1INH types I and II, HAE with plasminogen mutation, and HAE of unknown cause. Acquired C1 inhibitor deficiency is also captured in the broader angioedema dataset. Diagnostic evaluation includes C4, C1 inhibitor antigen, C1 inhibitor function, and C1q in specialized laboratories, but reimbursement is incomplete and functional or genetic testing may require international collaboration. Median diagnostic delay is reported as 10.2 years in a retrospective cohort and 13.9 years in a more recent prospective cohort. ODT with plasma-derived or recombinant C1 inhibitor and icatibant is registered and reimbursed. LTP with lanadelumab and berotralstat is reimbursed for patients aged at least 12 years, with special pathways for younger children. However, a major structural barrier remains: the National Health Insurance Fund covers only one reimbursed treatment category per patient, either ODT or prophylaxis, rather than both.

The Bulgarian model demonstrates both the power and the vulnerability of disease-specific expertise. A registry maintained for decades offers continuity and clinical memory, and modern prophylaxis is reimbursed for eligible patients. However, the absence of an updated national rare-disease plan and the rule that separates reimbursed ODT from prophylaxis may undermine guideline-consistent care. A patient receiving LTP still requires an emergency plan and access to rescue therapy for breakthrough or laryngeal attacks.

### 3.4 Croatia

Croatia has a public, mandatory, solidarity-based healthcare system funded through the Croatian Health Insurance Fund. A new National Commission for Rare Diseases was established in 2023 to develop and monitor a new national program; measures and outcomes have been defined, although a dedicated budget remains a challenge. Croatia reports approximately 120 individuals with HAE nationally, while BEACON materials specifically detail 98 adults and 18 minors, corresponding to approximately 3.1 per 100,000 people, with diagnostic delay ranging from 5 to 43 years and a median of 13 years. Croatian experience has contributed important clinical and genetic data, including reports of severe laryngeal disease, pediatric diagnostic challenges, and a recent nationwide adult survey (11–13). C1 inhibitor antigen and functional assays are available at seven HAE centers, while C1q testing has required referral abroad or special arrangements. SERPING1 testing is performed at University Hospital Center Zagreb, and broader HAE genetic testing is supported by collaboration with Slovenia; expansion of routine HAE-C1INH and HAE-nC1INH genetic diagnostics at Zagreb is planned. ODT includes icatibant and plasma-derived or recombinant C1 inhibitor, administered in hospital or at home. LTP options include attenuated androgens, tranexamic acid, lanadelumab, and berotralstat. HAE Hrvatska, affiliated with HAEi, supports education and outreach; continuing needs include targeted physician education, expanded diagnostic capacity, and improved prophylaxis access.

### 3.5 Greece

Greece reports a national HAE registry initiative that began in 2009 and has generated published outputs (14). The latest update includes 55 families and 185 diagnosed patients, with an estimated national total of 200–300 and an average diagnostic delay of 13 years. A structured expert network operates across the country, including two certified Angioedema Centers of Reference and Excellence (ACARE), one National Center of Excellence, a dedicated outpatient clinic for children and adolescents, and a 24-hour helpline for urgent clinical support. Complement diagnostics and genetic testing are available, and national work has strengthened molecular diagnosis and understanding of primary angioedema with normal C1 inhibitor (15, 16). A current national initiative aims to provide free genetic testing for patients and relatives, emphasizing cascade screening. Greece reports broad access to ODT, including intravenous C1 inhibitor concentrate and widespread self-administration of icatibant. LTP is available and reimbursed for modern options including lanadelumab, berotralstat, subcutaneous C1 inhibitor, and garadacimab, and self-administration training is encouraged by national guidance (17). Remaining needs include persistent diagnostic delay, awareness gaps among emergency and otorhinolaryngology clinicians, geographic barriers for islands and rural areas, and psychosocial support for families. The Greek HAE patient association, founded in 2021, is active in advocacy, education, and policy dialogue.

### 3.6 Romania

Romania has had a national HAE registry since 2006, hosted and managed by the national HAE/ACARE center in Sângeorgiu de Mureș, which coordinates diagnostic and treatment programs nationally. Romania also has an official National Rare Disease Plan within the National Strategy for Health 2024–2030, with dedicated funding allocation. The country reports 176 patients with HAE type I or II and one with HAE-nC1INH; some previously diagnosed patients are no longer followed in the national system because of migration. Mean diagnostic delay is 15.6 years based on 84 patients and is longer in adults than in children. C4 is covered by the public health system; C1 inhibitor antigenic and functional assays are available free of charge through a company-sponsored project; C1q testing is available only in private laboratories; and genetic testing is accessible via collaboration with other HAE centers or out-of-pocket payment. All HAE treatments are fully reimbursed, even for uninsured patients, and specific subcutaneous or intravenous treatments are approved for home administration. ODT includes plasma-derived C1 inhibitor, icatibant, and FFP. LTP includes intravenous or subcutaneous plasma-derived C1 inhibitor, lanadelumab, and berotralstat, while attenuated androgens are used by a small number of patients through procurement from abroad. Two HAEi-affiliated patient organizations support advocacy and education; priorities include genetic testing, maintaining full reimbursement, broader new-drug availability, and emergency-department education.

### 3.7 Serbia

Serbia has a mandatory public insurance system and a national rare-disease register operated by the Institute of Public Health “Dr Milan Jovanović Batut”, aligned with European minimum-dataset coding, including ORPHA and OMIM identifiers. Current reports describe 118 persons with HAE-C1INH, and the Rare Diseases Commission has approved treatment for 96 insured patients. Severe historical outcomes include two laryngeal deaths and emergency tracheotomy in some patients. A 2018 snapshot reported 80 patients and a median diagnostic delay of 12.5 years; Serbian investigators also described novel SERPING1 mutations and genotype-phenotype associations (18). Complement component 3 and 4 testing is widely available; C1 inhibitor antigen is available at multiple centers; C1q and C1 inhibitor function are offered at key institutions; anti-C1 inhibitor antibody testing is unavailable. Since 2021, rare-disease centers can send samples for nationally funded next-generation sequencing analysis at the Institute of Molecular Genetics and Genetic Engineering, University of Belgrade. Treatment has expanded from danazol and tranexamic acid to plasma-derived or recombinant C1 inhibitor, icatibant, lanadelumab, and berotralstat. In February 2026, the first patient started LTP with subcutaneous plasma-derived C1 inhibitor. Key needs are self-administration training, distribution and stock control, and supply continuity outside major centers. HAE Srbija, founded in 2016, is a full HAEi member.

### 3.8 Slovenia

Slovenia has a national rare-disease plan and a structured approach to rare-disease management, including registry-based monitoring and a national HAE center, which has been a certified ACARE center since 2019. The country reports 28 patients with HAE-C1INH and two with HAE related to factor XII mutation under follow-up. Historically, diagnostic delays were long, reaching up to 40 years in some cases, with a median of 13 years, and Slovenian investigators have reported nationwide genetic data including novel SERPING1 mutations (19). The national center has access to complement diagnostics and advanced genetic testing, covered by national health insurance for appropriate indications. More specialized molecular testing can be arranged domestically or through international collaboration. ODT includes intravenous plasma-derived or recombinant C1 inhibitor and icatibant, with home treatment covered. LTP includes intravenous C1 inhibitor, lanadelumab, and berotralstat for selected patients, while danazol remains in use only for a small number of long-standing cases. HAE Slovenia is linked to HAEi and highlights the continuing need for family screening and awareness, particularly for HAE-nC1INH. Slovenia's compact geography and specialist infrastructure support coordinated care, but the small absolute number of patients makes sustained clinical expertise, registry continuity, and access to novel diagnostics dependent on stable national commitment and cross-border collaboration.

### 3.9 Türkiye

Türkiye adopted a national rare-diseases plan in 2022 for the 2023–2027 period and established a Ministry of Health rare-diseases department. The Turkish Hereditary and Acquired Angioedema Registry records patients across 55 centers in 24 cities; BEACON reported 658 patients in this combined HAE/acquired angioedema registry, including HAE-C1INH, HAE-nC1INH, and acquired angioedema (20, 21). Estimated HAE prevalence is 1:10,000–1:50,000, with more than 7,500 undiagnosed individuals. Mean diagnostic delay for HAE-C1INH is 15.4 years (standard deviation 14.5; median 12; maximum 76). Complement and genetic testing are fully available and reimbursed at major referral centers such as Ege, Istanbul, and Hacettepe; other institutions rely partly on outsourcing. C1q and anti-C1 inhibitor autoantibodies are performed only at Ege. ODT includes hospital-use plasma-derived C1 inhibitor and home-use icatibant, both approved and reimbursed; recombinant C1 inhibitor is not approved. For prophylaxis, danazol and tranexamic acid are approved and covered, and 34% of patients still use danazol; lanadelumab and berotralstat are approved but not reimbursed. Compassionate or named-patient import pathways are used for unregistered drugs. HAÖDER, founded in 2015, supports awareness, access, and patients.

## 4 Assessment of policy and practice options and implications

Taken together, these country-level profiles show that regional variation in HAE care is shaped by a recurring set of policy and practice domains rather than by isolated national circumstances. The thematic analysis below, therefore, examines the organizational, diagnostic, therapeutic, and implementation factors that most consistently determine access to care across the included Balkan Peninsula-area health systems.

### 4.1 Governance, specialist networks, and case finding

HAE care across the included Balkan Peninsula–area health systems sits at the intersection of rare-disease policy and specialist allergy/immunology practice. Countries in the region have adopted different organizational models. Some have identifiable rare-disease plans, registries, and specialist networks that create a recognizable administrative pathway for diagnosis and treatment. Others rely more heavily on the initiative of individual clinicians, informal referral patterns, or entity-level frameworks that do not consistently translate into national access. Table 1 illustrates this gradient: Greece, Romania, Serbia, Slovenia, Türkiye, and Bulgaria each have some combination of rare-disease governance, registry structure, recognized centers, or mature patient representation, whereas Albania and Bosnia and Herzegovina face more fragile or uneven governance environments.

This is important because HAE exemplifies how policy structures shape clinical outcomes. Concentrating specialist expertise isn't necessarily inefficient. Indeed, for rare diseases, centralization can work if the hub is accessible, capable of diagnosing, and connected to satellite services. Problems occur when such concentration lacks clear referral pathways, transport options, sample routing, teleconsultation, or home-treatment facilities. In those cases, specialist excellence in a city center may coexist with practical barriers for patients in islands, mountains, rural areas, or cross-border regions.

The region's published literature shows that scientific and clinical expertise already exists. Croatian reports describe severe laryngeal attacks in families with SERPING1 mutations and highlight pediatric diagnostic and prophylactic challenges (11, 12). Greek registry and national consensus work show the benefits of national coordination, while molecular studies from Greece, Serbia, Slovenia, and Türkiye demonstrate robust diagnostic and research capacity (14–21). This is an important policy point: the main regional deficit is not the absence of expertise, but the uneven system translation of expertise into accessible care.

Underdiagnosis remains a common concern. Several countries report far fewer diagnosed patients than expected within accepted prevalence ranges, even in the presence of expert centers. That pattern suggests missed family screening, limited recognition of disease outside referral centers, or delayed access to confirmatory testing. Family-based case finding is particularly valuable in HAE because each confirmed index case offers an immediate opportunity to screen relatives at a comparatively low marginal cost. In policy terms, family screening is not an optional enhancement but one of the most efficient rare-disease case-ascertainment tools available.

Different organizational options carry different implications. A country may invest in many nominal centers, as in Türkiye, or maintain a smaller number of highly specialized hubs, as in Slovenia or Romania. Neither model is inherently superior. What matters is whether the model supports early recognition, reliable testing, rapid access to ODT, and continuity of follow-up. Policy evaluation should therefore focus less on the raw count of centers and more on what those centers can do, how accessible they are, and whether they are embedded in a referral network that non-specialists understand.

### 4.2 Diagnostic pathways and laboratory equity

The diagnostic pathway is the primary source of regional inequality. At a minimum, patients with suspected HAE-C1INH need access to C4, C1 inhibitor antigenic level, and C1 inhibitor functional testing. C1q is particularly useful when acquired angioedema is in the differential diagnosis, whereas anti-C1 inhibitor antibodies and genetic testing are relevant in selected cases (1, 2, 4). In principle, these tests are neither conceptually novel nor technologically exotic. In practice, however, their availability, affordability, and logistical accessibility vary considerably across the included health systems.

C4 testing is generally regarded as the most accessible investigation throughout the region; however, more comprehensive diagnostic assessments are less consistently available. Several participating countries indicate that testing for C1 inhibitor function, C1q, or genetic analysis may be conducted locally but may not be publicly reimbursed, may be restricted to specific laboratories, or may require private payment or international sample shipment. Although these distinctions may seem administrative in nature, they hold significant clinical implications. A diagnostic pathway dependent on self-financing or *ad hoc* collaborations is susceptible to delays, inequities, and interruptions. This situation is particularly critical for children, relatives of index cases, and patients living far from tertiary hospitals.

Greek molecular studies illustrate the practical value of expanded diagnostic capacity. Targeted next-generation sequencing has improved molecular diagnosis of HAE due to C1 inhibitor deficiency, and research on primary angioedema with normal C1 inhibitor levels has clarified the genetic heterogeneity of non-classical presentations (15, 16). In practice, such capacity supports subtype confirmation, family screening, and decisions about when reference-laboratory testing is needed. These advances do not mean that every country needs a fully independent molecular program. They do, however, show that policy systems should guarantee access to advanced testing when clinically indicated. For smaller countries, a cross-border or regional reference-laboratory arrangement may be more realistic and more cost-effective than duplicating low-volume assays in every setting.

The persistence of long diagnostic delays across the region indicates that laboratory availability alone is insufficient. Many country reports still describe average delays of roughly 10–15 years, with some patients waiting substantially longer. Such delays imply repeated misclassification as “allergic” angioedema, exposure to ineffective antihistamines, corticosteroids, or epinephrine, and missed opportunities for family screening and prevention. The delay problem is therefore both educational and structural: clinicians must suspect the diagnosis and have an uncomplicated way to confirm it.

Two policy options are evident in the regional landscape. The first is a distributed model in which multiple hospitals can order and interpret core complement tests. The second is a hub-and-spoke model in which samples are routed to one or a few reference laboratories with defined turnaround times and referral criteria. Either model can work, but both require governance. Without reimbursement, logistics, and feedback loops, the distributed model becomes uneven, and the hub-and-spoke model becomes exclusionary. A pragmatic regional priority would be to define a minimum diagnostic package, specify where each assay should be performed, and establish a written algorithm for when samples are referred nationally or internationally.

The policy implication is straightforward: diagnostic equity should be treated as a reimbursable service pathway, not as a passive byproduct of a laboratory's existence. In rare diseases, the most important question is often not “Is the test available somewhere?” but “Can a non-specialist order it quickly enough for the patient who needs it?”

### 4.3 On-demand treatment as a minimum standard

Access to effective on-demand treatment is the clearest indicator of whether an HAE system protects patients from immediate harm. International guidance recommends treating attacks as early as possible and ensuring patients have access to disease-specific rescue therapy rather than relying on antiallergic medication or delayed hospital treatment (2). Public-facing HAEi materials emphasize that access to effective modern treatment, including self-administration where permitted, improves quality of life (9). In other words, a system cannot be considered modern if a patient with a known diagnosis still depends primarily on emergency transport and fresh frozen plasma for routine attack management.

The country comparison suggests that effective ODT is available in most participating settings, but its form and practical accessibility vary markedly. Some countries report routine availability of plasma-derived or recombinant C1 inhibitor and/or icatibant, while others continue to rely partly on fresh frozen plasma or on center-based access to specific therapy. A recurring regional phenomenon is “pseudo-access”: a product is approved or technically available, but patients cannot count on it being stocked nearby, reimbursed for routine use, or available for home administration. From the patient's perspective, that difference is clinically indistinguishable from non-access when an attack occurs.

Hospital-only rescue pathways are particularly problematic. They may appear safe from an administrative standpoint because they confine prescribing and product handling to supervised settings, but they cause delays that conflict with HAE pathophysiology and guideline recommendations for early treatment (2). They also create obvious geographic inequities. A patient in an urban area near a tertiary hospital experiences a different therapeutic reality than a patient on an island, in a mountainous district, or in a rural municipality several hours from the nearest stocked center.

The policy discussion should shift focus from just which ODT product is listed nationally to whether patients can access it promptly. Countries permitting self-administration or home stock of ODT effectively expand a vital window for patient care and reduce the effects of geographic disparities on outcomes. Conversely, countries relying on tertiary centers may control use more strictly, but this approach often compromises the promptness and equity of access. For a rare disease with unpredictable attacks, including potentially life-threatening laryngeal attacks, this trade-off warrants clear and thorough review.

Another implementation challenge is the incorrect belief that ODT is less important when prophylaxis is available. Clinical practice does not support this view. Patients on prophylaxis still need rescue treatment for breakthrough attacks, for diagnostic uncertainty early in treatment, or when delays could be dangerous. A reimbursement system that covers prophylaxis but restricts rescue therapy, or demands a choice between them, is inconsistent with clinical needs. Therefore, the minimum requirement should be access to specific ODT for every diagnosed patient, with the method of access tailored to local conditions but the principle remaining non-negotiable.

### 4.4 Long-term prophylaxis and reimbursement design

LTP has become a defining fault line between countries that can fully implement contemporary HAE guidelines and those that cannot. Modern recommendations place prophylaxis within an individualized risk-benefit framework rather than a narrow numerical threshold based solely on attack counts (2). The relevant clinical question is whether recurrent or severe attacks, laryngeal risk, occupational exposure, travel patterns, pregnancy planning, psychosocial burden, or patient preference justify an intervention that reduces attack frequency and unpredictability. This patient-centered approach requires a reimbursement system that can accommodate clinical nuance.

The regional picture is heterogeneous. Greece and Romania report broad access to modern prophylactic options, while Bulgaria and Serbia have made important progress but still face category-specific or administrative constraints in some cases. Croatia and Slovenia appear comparatively well aligned with guideline-era practice for small-country settings. Türkiye reports availability of modern prophylactic therapies but incomplete reimbursement for some, with traditional agents such as danazol and tranexamic acid still playing a larger role in routine care than would be preferred in fully updated systems. Bosnia and Herzegovina and Albania remain much more constrained, with modern prophylaxis either absent, very limited, or dependent on exceptional arrangements.

These differences are important because reimbursement strategies are inherently biased. When modern prophylaxis is not covered, clinicians and patients might depend more on less effective or unacceptable options like attenuated androgens or antifibrinolytics. Conversely, if a country reimburses a modern medication but restricts access through strict bureaucratic criteria, repeated approvals, or rigid category rules, the official benefit may seem generous while the actual implementation remains fragile. Therefore, policy analysis should consider not just whether a medicine is reimbursed, but also how that reimbursement is put into practice.

A related issue is the use of clinical trials or named-patient pathways as informal access routes. Bulgaria and Türkiye, for instance, have gained exposure to innovative therapies through trial activity or transitional availability. Such participation is valuable for scientific advancement and may offer early access to some patients, but it is not an acceptable substitute for stable policy. Trial access remains selective, temporary, and dependent on sponsor activity. A comprehensive reimbursement system should incorporate evidence-based innovations into routine care, rather than viewing research participation as a workaround for standard access (20).

The most clinically consistent policy model is one that recognizes three principles: rescue therapy must be accessible to all diagnosed patients; prophylaxis must be individualized rather than restricted by simplistic thresholds; and treatment options should be flexible throughout the disease course, including escalation, de-escalation, and adjustments for different life stages. This approach aligns funding with actual clinical needs. Anything less than this could perpetuate inequality within the benefits system.

### 4.5 Home treatment, self-administration, and emergency readiness

If ODT and LTP serve as the pharmacologic foundations of modern HAE management, then home treatment and emergency preparedness constitute the operational foundations. Early treatment is most effectively provided when the patient or caregiver can administer therapy immediately, without delays for hospital arrival, and when emergency departments that see the patient can identify bradykinin-mediated swelling and respond appropriately (2, 17). However, these capabilities vary across the included health systems.

Some countries have integrated home treatment and self-administration into their standard practices, especially for icatibant and, in certain cases, C1 inhibitor products. Others still limit parts of treatment to hospital settings, face prescribing or dispensing delays, or lack comprehensive training programs for patients and caregivers. This is especially important in regions where geographic factors cause treatment delays. In regions with islands, mountainous terrain, or centralized urban areas, home treatment is more than just convenient; it is a critical equity measure that reduces reliance on travel and infrastructure.

Emergency preparedness goes beyond stockpiling medications. Patients should have personalized emergency plans, documentation for emergency personnel, and clear procedures for managing laryngeal symptoms, peri-procedural issues, and pregnancy-related concerns. Non-specialists in fields such as emergency medicine, otolaryngology, gastroenterology, dermatology, pediatrics, obstetrics, anesthesia, and primary care need to recognize potential HAE symptoms and understand why corticosteroids and antihistamines may be ineffective for bradykinin-mediated attacks. National consensus documents, such as the recent Greek guidance, can help translate international recommendations into locally applicable algorithms (17).

Pediatric and life-course considerations highlight the importance of operational flexibility. Croatian pediatric experience reveals prophylactic and diagnostic challenges in children (12), while Turkish studies on quality of life, sleep, and family planning demonstrate how HAE impacts routine functioning, reproductive choices, and long-term psychosocial adaptation (23–25). International surveys of physicians confirm that unmet needs remain, even where effective treatments are available, especially when education, access, or patient-centered service design are lacking (26). Special circumstances such as pregnancy, breastfeeding, procedures, school attendance, or travel require proactive planning instead of reactive responses. Systems designed solely around adult tertiary-clinic visits are unlikely to address these needs adequately.

From a policy perspective, self-administration programs are often low-hanging fruit. Once regulatory, prescribing, and training barriers are addressed, they can reduce emergency utilization, improve patient confidence, and partly offset inequalities generated by specialist-center concentration. Countries that have not yet normalized home treatment should view it as a practice redesign priority rather than as an optional future refinement.

### 4.6 Registries, data governance, and quality monitoring

Registries deserve a central place in HAE policy because they turn rare, scattered cases into accessible health-system data. A registry can document diagnostic delays, genotype-phenotype distributions, treatment utilization, outcomes, emergency use, prophylaxis uptake, and geographic concentrations of unmet needs. It can also support family screening, forecast medication demand, support health technology assessment, and evaluate whether reimbursement decisions enhance care in practice. In the context of rare diseases, these functions are particularly valuable because anecdotal impressions may otherwise dominate planning.

The regional picture ranges from mature or long-standing registries to informal working lists or absence of a formal data structure. Romania reports registry activity dating back to 2006, Greece has published initial national registry results, Serbia and Slovenia have nationally coordinated datasets, and Bosnia and Herzegovina has at least a developing registry function despite broader governance constraints (14, 18, 19). The existence of a registry, however, should not be equated with full analytic maturity. Some registries primarily count patients, while others support longitudinal follow-up and policy assessment.

The experience of the global HAE registry demonstrates how harmonizing core variables can enable meaningful comparisons while respecting local differences (27). The Balkan Peninsula does not need to replicate any particular external model entirely. Instead, it would benefit from establishing a consensus on a minimum dataset for routine clinical and policy purposes, including diagnosis and subtype; age at symptom onset and diagnosis; family history and relatives screened; available ODT; home-treatment status; prophylaxis use; major breakthrough attacks; laryngeal events; emergency visits or hospitalizations; and, where possible, patient-reported impact indicators.

Effective registry management is essential to establishing credibility. For a registry to be sustainable, it must have institutional ownership, ensure data protection, provide clear definitions, and include a plan for data use. If registry data are not linked to service redesign, procurement, or quality improvement, clinicians and patients may view data entry as an administrative burden rather than a valuable tool. Therefore, the strongest policy argument for investing in registries is their practical value: they should provide answers to questions that matter to patients, clinicians, and payers.

A data framework aligned with the regional BEACON standards could be extremely useful. Even if each country maintains its own registries, using harmonized indicators would enable comparisons of progress in areas like time to diagnosis, family screening success, access to ODT at home, prophylaxis adherence, and emergency preparedness. This shift would assist the region in moving from basic mapping to monitoring quantifiable quality enhancements.

### 4.7 Patient organizations, advocacy, and shared decision-making

Patient organizations are not peripheral to HAE care; in many countries, they are part of the delivery infrastructure. HAEi materials have emphasized that disparities in disease recognition, treatment approval, and reimbursement remain major obstacles across the included health systems, even where scientific understanding and international guidance have advanced (9). In the current regional review, patient-organization input helped identify recurring implementation problems that clinicians also described: insufficient awareness outside expert centers, uneven physician knowledge, delayed access to modern therapy, and weak political prioritization of rare diseases.

This convergence is important because policy recommendations become more robust when both clinicians and patients agree on the main bottlenecks. It reduces the chance that the analysis simply reflects the interests of one group. Additionally, it serves as a reminder to policymakers that evaluating HAE outcomes should go beyond just attack frequency or hospital visits. Recent research highlights significant impacts on patients' quality of life, sleep, and family planning, further supporting the idea that access to treatment influences everyday life participation as much as emergency rescue efforts.

Patient organizations play a crucial role in addressing practical issues often overlooked by formal health systems. They provide accessible disease information, assist with understanding reimbursement policies, encourage family testing, facilitate peer exchange on home treatment, and highlight the real-world impacts of stockouts or administrative delays. In small countries, they can be the only consistent structure that ensures continuity when expert clinicians retire, migrate, or face overstretched workloads.

The regional setting indicates that advocacy capacities vary significantly across areas. Established patient groups connected to HAEi can help speed up diagnosis and reimbursement discussions. In contrast, where organizations are weak or just forming, patients rely more heavily on individual clinicians' efforts. The North Macedonian case illustrates this: even if a country isn't fully represented in the comparative policy overview, it can still possess valuable regional clinical experience and an active patient community (22). Consequently, a regional network should prioritize strengthening patient organizations as an essential policy goal.

Shared decision-making is especially important. In HAE, factors like the route of administration, speed of access, tolerability, unpredictability of attacks, occupational exposure, pregnancy planning, and acceptance of residual risk vary significantly among patients. Policies that offer nominal access but restrict personalized treatment choices may meet reimbursement criteria but fall short of true patient-centered care. Engaging patients in guideline development, registry governance, and reimbursement discussions is an effective way to ensure policies better reflect the real disease experience.

### 4.8 Country-level implementation trajectories and policy implications

The countries included in this review can be understood as following different implementation paths rather than forming a straightforward ranking. A first group consists of relatively mature systems where several components—such as specialist care, diagnostics, registry activities, and access to rescue and prophylactic treatments—are already well coordinated for many patients. Greece and Slovenia serve as good examples, and Romania also shows significant system maturity despite ongoing issues with underdiagnosis and geographic disparities. These systems should shift their focus from merely ensuring basic availability to enhancing internal equity, expanding family screening efforts, implementing registry-based quality monitoring, and optimizing care throughout patients' lives.

A second group includes countries that have shown significant progress but still encounter notable gaps between policy and everyday practice. Bulgaria, Croatia, Serbia, and Türkiye generally fit this group, though their situations vary. Bulgaria has modern therapeutic exposure and active advocacy but continues to face issues related to diagnostic reimbursement and sustainability. Croatia and Serbia possess strong scientific and clinical expertise with relatively good treatment access, yet they need ongoing efforts to improve dispersion, transition, and ensure fully coherent access rules. Türkiye features a large network of specialists and a registry, but still has reimbursement gaps for certain modern prophylaxis and restrictions on home or hospital use for some products. These nations are suitably situated for targeted policy adjustments rather than comprehensive redesign.

A third group includes settings where the core HAE pathway is still somewhat fragile. Albania and Bosnia and Herzegovina exemplify this pattern differently. Albania has a functioning tertiary center and some access to modern therapy but faces limited reimbursement for diagnostics and a restricted access base. Bosnia and Herzegovina has documented patient cases, some diagnostic capacity, and approved modern treatments; however, reimbursement and routine access are still weak. In both countries, the immediate policy priority is to establish a basic package: confirmatory diagnostics, access to specific ODT, clear referral pathways, and a practical registry system.

This trajectory-focused perspective offers two main benefits. First, it prevents framing regional comparisons solely as a matter of reputation. Second, it highlights that each country requires different subsequent actions. Smaller nations might gain the most from hub-and-spoke care, cross-border laboratory collaborations, and regional training programs. Conversely, larger healthcare systems could benefit more from decentralization, stabilized procurement, and harmonized practices across institutions. A regional network can support both approaches by sharing templates, training modules, registry variables, and reimbursement strategies, all without insisting on a single organizational structure.

The region's published output also holds symbolic policy significance. When a country contributes molecular, registry, consensus, pediatric, or cohort data to the international HAE literature, it demonstrates that local expertise can produce evidence, participate in networks, and adopt innovations (11–22). This matters in reimbursement discussions because payers and ministries are more likely to view HAE as a legitimate policy priority when domestic clinicians and researchers can document both the disease's burden and their practical capacity to deliver care. In essence, regional scholarship is not just an academic achievement; it can also enhance the credibility of implementation.

The policy implication is that this regional health-system comparison serves as a source of transferable practices. Established systems in one country can facilitate practical improvements in another, especially when the main obstacle is organizational rather than scientific. Table 1 is intended to support exactly that type of benchmarking and knowledge sharing.

## 5 Actionable recommendations

The thematic analysis above leads to a practical conclusion: disparities in HAE care will narrow only if each stakeholder group addresses the barriers within its own area of responsibility. The recommendations below are therefore organized by stakeholder group. They aim to translate the preceding analysis into practical actions related to financing, service organization, emergency preparedness, data systems, and patient support.

### 5.1 Priorities for ministries of health and payers

**Recommendation 1: Define a minimum national HAE benefit package.** Every diagnosed patient should have a guaranteed pathway to core diagnostic testing, disease-specific on-demand treatment, and specialist review for prophylaxis when clinically indicated. The benefits package should clearly differentiate among approval, reimbursement, and practical access, and it should not consider HAE sufficiently covered solely because a product has been licensed.
**Recommendation 2: Eliminate clinically incoherent reimbursement rules.** Reimbursement pathways should neither force a choice between prophylaxis and rescue therapy nor rely on step-therapy approaches that delay access to modern treatment due to administrative rather than clinical concerns. Eligibility criteria should be clear, evidence-based, and adaptable as new evidence and local experiences emerge.
**Recommendation 3: Stabilize procurement and local stock pathways.** National or hospital tenders should ensure that rescue therapies are physically accessible to patients in a timely manner. Regular stockouts, reliance on a single site, or unclear importation rules should be regarded as safety issues, not just administrative inconveniences.

### 5.2 Priorities for specialist centers and professional societies

**Recommendation 4: Publish a national referral and testing algorithm.** Each country should establish clear procedures for how a suspected HAE patient progresses from initial clinical suspicion to confirmatory testing and specialist consultation. This process should specify criteria for family screening, identify where key assays are conducted, and detail how samples are referred when advanced testing is centralized.
**Recommendation 5: Standardize written emergency plans and patient-held documentation.** Every diagnosed patient should hold a specialist review with a clear emergency plan that non-specialists can understand. Professional societies can assist by translating international guidelines into concise, locally applicable algorithms for emergency medicine, ENT, gastroenterology, obstetrics, pediatrics, and anesthesia.
**Recommendation 6: Expand structured home-treatment and self-administration training.** Where it is clinically appropriate and feasible within regulations, specialist centers should educate patients and caregivers on the proper use of ODT at home. This is especially important in geographically dispersed areas and should be monitored as a service-quality indicator instead of relying solely on individual clinician choice.

### 5.3 Priorities for emergency-care systems and non-specialist clinicians

**Recommendation 7: Treat emergency recognition of HAE as a cross-disciplinary competency.** HAE cannot be managed safely if disease knowledge remains confined to expert clinics. Hospitals and emergency systems should incorporate short HAE modules into routine education, emphasizing when bradykinin-mediated swelling is likely, what therapies are ineffective, and when airway risk requires immediate action.
**Recommendation 8: Create institution-level HAE response protocols.** Emergency departments and peri-procedural settings should be aware of the location of HAE rescue medication, the authorized personnel for its use, and the circumstances requiring specialist consultation. For smaller hospitals, the protocol should incorporate teleconsultation or referral support rather than assuming in-house expertise.

### 5.4 Priorities for registry and data-governance stakeholders

**Recommendation 9: Establish or strengthen a core HAE registry dataset.** Even when a full disease-specific registry is not immediately feasible, countries should capture a minimum common dataset including diagnosis, subtype, time to diagnosis, family screening, ODT access, home-treatment status, prophylaxis exposure, major attacks, and patient-reported impact. Registry data should be reviewed regularly with a policy purpose in mind.
**Recommendation 10: Establish regional indicators for benchmarking purposes.** A BEACON-aligned network could evaluate countries using a limited set of feasible indicators: median diagnostic delay, proportion of diagnosed patients with ODT at home, proportion with a written emergency plan, proportion of index cases with family screening, proportion of prophylaxis users who also have rescue therapy, and registry completeness. Standardized indicators would clearly show progress and facilitate more convincing discussions with payers and ministries.

### 5.5 Priorities for patient organizations and regional collaboration

**Recommendation 11: Formalize patient-organization involvement in policy processes.** Patient groups should be involved proactively rather than only being consulted after reimbursement disputes occur. They need to be included in activities such as guideline development, registry governance, educational initiatives, and assessing the real-world effectiveness of reimbursement policies.
**Recommendation 12: Build a practical regional collaboration platform.** A BEACON-aligned regional HAE network should share referral maps, diagnostic pathways, patient education materials, self-administration resources, and advocacy tools. Countries with mature systems can mentor those building core pathways, while smaller countries can pool expertise through cross-border laboratory agreements or joint educational initiatives.
**Recommendation 13: Separate research participation from access policy.** Clinical trials should be encouraged but not allowed to substitute for routine treatment access. When innovation enters a country via research activity, stakeholders should leverage that experience to develop sustainable adoption strategies instead of making patients dependent on temporary solutions.

## 6 Discussion

The primary conclusion of this review is that HAE care in the examined health systems across the Balkan Peninsula should not be considered limited due to lack of scientific knowledge. The region has active clinicians, published cohorts, genetic expertise, patient organizations, and access - at least somewhere in the system - to many of the diagnostic and therapeutic tools required for modern care (2, 11–22). Persistent inequities arise because these elements are not yet consistently aligned within national health systems. Regulatory approval without reimbursement, reimbursement without practical access, center-based expertise without clear referral pathways, and registry intentions without usable data all create avoidable gaps in care (7, 27).

This observation has two important implications. First, it shifts the policy debate from focusing on rarity to emphasizing implementation. HAE is rare, but the challenges discussed in this review are not exclusive to HAE; they are common issues faced by rare diseases, including centralized services, small patient populations, fragmented budgets, and limited policy attention. HAE can therefore serve as a valuable indicator for understanding how health systems handle time-sensitive rare diseases overall. Additionally, it indicates that focused organizational reforms can lead to significant improvements without the need for extensive structural changes. Implementing clear diagnostic pathways, improved stock logistics, self-administration training, establishing minimum datasets for registries, and promoting formal patient participation are practical interventions (2, 7, 27).

A further implication is that equity in HAE should be operationally defined by real-world access to care, rather than by declarative policy commitments alone. A country does not provide equitable care because it has a listed medicine, a named expert center, or a draft registry. It provides equitable care when a patient can obtain an accurate diagnosis without delay, receive rescue treatment rapidly, access prophylaxis when clinically indicated, navigate emergency care safely, and participate in decisions that affect everyday life (2, 5–7, 26). This functional definition is especially important in regions where formal structures may exist but do not consistently translate into equitable care in practice.

The review also highlights the need to align policy design with country scale. Smaller countries do not necessarily need to reproduce every advanced diagnostic or therapeutic function domestically, provided that reliable cross-border or centralized referral pathways are in place. Larger countries with multiple centers, by contrast, require internal harmonization to ensure that geographic variation does not undermine national policy. Regional collaboration should therefore be viewed not as a substitute for national responsibility, but as a mechanism to reduce duplication, accelerate training, and strengthen political leverage for investment in rare diseases.

A regional approach may also help address difficult national policy questions in a less political way. In small and middle-sized health systems, rare-disease policy often advances only when local stakeholders can demonstrate that proposed reforms have already been achieved in neighboring countries with comparable resources. This regional comparison is therefore useful. It can show that home treatment, patient registries, family screening, and more coherent reimbursement policies are not unrealistic goals taken from very different health systems, but practical steps that are already being applied in the same broader region. This type of realistic comparison may be more convincing than simply referring to guideline recommendations.

Several limitations should be acknowledged. This analysis is descriptive and comparative, not systematic. Some country-level details on implementation were based on expert and advocacy-informed materials prepared for BEACON, rather than on independently audited public datasets. In addition, access arrangements may change rapidly because of changes in reimbursement rules, tenders, or supply chains. Countries for which sufficiently detailed and comparable information was not available could not be included in the full table. For these reasons, this review should be interpreted as a policy map, rather than as a definitive epidemiological census. Even with these limitations, the consistency between published literature, expert reports, and patient perspectives supports the conclusion that the disparities described are real, repeated across settings, and potentially actionable.

Future work should build on this mapping exercise in two ways. First, countries should make policy and registry information more publicly available and easier to retrieve, so that future reviews depend less on expert reporting. Second, the BEACON network, or a similar regional collaboration, should move beyond description and start measuring progress through shared indicators and regular updates (27). In rare diseases, visibility is itself a form of policy power.

In conclusion, HAE care across selected health systems in the Balkan Peninsula area shows substantial policy and practice variation despite a shared regional knowledge base and increasing availability of effective therapy. The most urgent next step is not another description of the disease but a coordinated implementation agenda built around minimum standards for diagnostics, rescue treatment, prophylaxis evaluation, home therapy, emergency preparedness, registry use, and patient partnership. If the region can translate its existing expertise into aligned policy and practice, many of the current inequities are likely to be preventable.
