# Supplementary material for: Hereditary angioedema care across selected health systems in the Balkan Peninsula area: policy gaps, practice variation, and actionable recommendations
Source: Front Allergy. 2026 Jul 20;7:1871835. doi: 10.3389/falgy.2026.1871835 (PMC13430603; doi:10.3389/falgy.2026.1871835)
Supplement: Supplementary file 1 [file Datasheet1.docx]

# Supplementary Data Sheet 1. PubMed bibliometric snapshot: search strategy and reproducibility details

## Objective

To provide a transparent, reproducible bibliometric snapshot of the representation of Balkan Peninsula area investigators in PubMed-indexed hereditary angioedema (HAE) literature, as contextual background for the BEACON initiative.

## Database and search interface

PubMed (National Library of Medicine), searched via the PubMed web interface.

## Date of search (retrieval date)

12 September 2025.

## Core concept and field selection

The HAE concept was represented by the component terms "hereditary" and "angioedema" in the Title/Abstract field. This approach was chosen to replicate the pragmatic bibliometric snapshot discussed during BEACON and is intended as a contextual indicator rather than an exhaustive bibliometric review.

## Time window

Publications dated through 12 September 2025.

## Definition of regional co-authorship for the bibliometric snapshot

Regional co-authorship was operationalized as at least one author affiliation containing one of the predefined country terms used for the BEACON bibliometric frame.

## A) Base query: PubMed-indexed HAE publications (global)

Query A1 (global HAE set)

("hereditary"[Title/Abstract]) AND ("angioedema"[Title/Abstract]) AND ("2000/01/01"[Date - Publication] : "2025/09/12"[Date - Publication])

Data extracted from PubMed result counts: global HAE publications since 2000, 2,691.

## B) Balkan Peninsula area co-authorship (overall)

Affiliation filter (Balkan Peninsula area)

(Albania[ad] OR "Bosnia-Herzegovina"[ad] OR "Bosnia and Herzegovina"[ad] OR Bulgaria[ad] OR Croatia[ad] OR Cyprus[ad] OR Greece[ad] OR Kosovo[ad] OR Moldova[ad] OR Montenegro[ad] OR "North Macedonia"[ad] OR Macedonia[ad] OR Romania[ad] OR Serbia[ad] OR Slovenia[ad] OR Turkey[ad] OR Türkiye[ad])

Query B1 (HAE plus Balkan Peninsula area co-authorship)

("hereditary"[Title/Abstract]) AND ("angioedema"[Title/Abstract]) AND ("2000/01/01"[dp] : "2025/09/12"[dp]) AND (Albania[ad] OR "Bosnia-Herzegovina"[ad] OR "Bosnia and Herzegovina"[ad] OR Bulgaria[ad] OR Croatia[ad] OR Cyprus[ad] OR Greece[ad] OR Kosovo[ad] OR Moldova[ad] OR Montenegro[ad] OR "North Macedonia"[ad] OR Macedonia[ad] OR Romania[ad] OR Serbia[ad] OR Slovenia[ad] OR Turkey[ad] OR Türkiye[ad])

Data extracted from PubMed result counts: HAE publications with Balkan Peninsula area co-authorship, 186 (7%).

## C) Clinical trial/randomized controlled trial subset

Query C1 (global HAE clinical trial/RCT publications)

("hereditary"[Title/Abstract]) AND ("angioedema"[Title/Abstract]) AND ("2000/01/01"[dp] : "2025/09/12"[dp]) AND ("clinical trial"[pt] OR "randomized controlled trial"[pt])

Query C2 (clinical trial/RCT publications with Balkan Peninsula area co-authorship)

("hereditary"[Title/Abstract]) AND ("angioedema"[Title/Abstract]) AND ("2000/01/01"[dp] : "2025/09/12"[dp]) AND ("clinical trial"[pt] OR "randomized controlled trial"[pt]) AND (Albania[ad] OR "Bosnia-Herzegovina"[ad] OR "Bosnia and Herzegovina"[ad] OR Bulgaria[ad] OR Croatia[ad] OR Cyprus[ad] OR Greece[ad] OR Kosovo[ad] OR Moldova[ad] OR Montenegro[ad] OR "North Macedonia"[ad] OR Macedonia[ad] OR Romania[ad] OR Serbia[ad] OR Slovenia[ad] OR Turkey[ad] OR Türkiye[ad])

Data extracted from PubMed result counts: global HAE clinical trial/RCT publications, 162; clinical trial/RCT publications with Balkan Peninsula area co-authorship, 17 (10.5%).

## D) Guideline/consensus subset

Query D1 (global HAE guideline/consensus publications; title filter)

("hereditary"[Title/Abstract]) AND ("angioedema"[Title/Abstract]) AND ("2000/01/01"[dp] : "2025/09/12"[dp]) AND (guidelines[ti] OR consensus[ti])

Query D2 (guideline/consensus publications with Balkan Peninsula area co-authorship)

("hereditary"[Title/Abstract]) AND ("angioedema"[Title/Abstract]) AND ("2000/01/01"[dp] : "2025/09/12"[dp]) AND (guidelines[ti] OR consensus[ti]) AND (Albania[ad] OR "Bosnia-Herzegovina"[ad] OR "Bosnia and Herzegovina"[ad] OR Bulgaria[ad] OR Croatia[ad] OR Cyprus[ad] OR Greece[ad] OR Kosovo[ad] OR Moldova[ad] OR Montenegro[ad] OR "North Macedonia"[ad] OR Macedonia[ad] OR Romania[ad] OR Serbia[ad] OR Slovenia[ad] OR Turkey[ad] OR Türkiye[ad])

Data extracted from PubMed result counts: global HAE guideline/consensus publications identified by title filter, 52; guideline/consensus publications with Balkan Peninsula area co-authorship, 5 (10%).

## Notes on interpretation and limitations

This bibliometric snapshot is not a systematic review and was not designed to capture all HAE-related literature exhaustively. Counts may change over time as PubMed indexing is updated, and affiliation-based searches may miss relevant regional contributions, particularly in records with incomplete affiliation information.

# Supplementary Data Sheet 2. Policy-mapping framework and operational definitions

## Purpose

To document the domains, labels, and decision rules used to harmonize country-level information for Table 1.

## Country coverage

The comparative policy map includes Albania, Bosnia and Herzegovina, Bulgaria, Croatia, Greece, Romania, Serbia, Slovenia, and Türkiye. Countries were included when sufficiently detailed and comparable system-level material was available for synthesis.

## Data domains extracted

Rare-disease governance; specialist network or centers; estimated and diagnosed patient counts; diagnostic delay; access to C4, C1 inhibitor antigen, C1 inhibitor function, C1q, anti-C1 inhibitor antibodies, and genetic testing; on-demand treatment; long-term prophylaxis; home treatment/self-administration; registry structure; patient organizations; innovation/clinical trial activity; and main reported implementation gap.

## Operational definition: approved

A medicine has marketing authorization or equivalent regulatory approval in the country, but this does not necessarily imply reimbursement, routine stock, or home use.

## Operational definition: available

A medicine or test can be obtained in practice by at least one recognized route within the country, including hospital stock, named-patient access, project-supported access, or center-based use. Availability does not imply affordability or equitable geographic access.

## Operational definition: reimbursed or covered

Public or mandatory insurance funding is reported for the medicine or test under routine or defined clinical circumstances. Partial reimbursement, exceptional approval pathways, or project-based access were not treated as equivalent to unrestricted routine coverage.

## Operational definition: home treatment or self-administration

The patient or caregiver can keep treatment outside the hospital and administer it after appropriate training, or can access a formally organized home-treatment pathway. A product that is only dispensed or administered in hospital was not classified as home treatment.

## Operational definition: registry

A registry label indicates a national, integrated, or working disease-specific data structure used for HAE case capture or follow-up. Informal case lists without an identifiable registry function were not classified as a formal registry unless country contributors explicitly described them as such.

## Operational definition: specialist center

A specialist center was counted when country contributors identified a hospital, unit, or network site with recognized responsibility for HAE diagnosis or management. The count reflects the reporting framework used in the country material and should not be interpreted as a formal accreditation count unless explicitly stated.

## Estimated patient count

Expected patient numbers reflect country-report estimates or working-table estimates informed by prevalence assumptions and local experience. They are presented to illustrate probable underdiagnosis and should not be interpreted as prevalence studies.

## Key gap

The key gap field summarizes the implementation problem most strongly emphasized by country contributors at the time the working table was finalized. It is intended to guide policy interpretation rather than to rank countries.

## Interpretive caution

Country situations may change as tenders, reimbursement decisions, or service organizations evolve. Table 1 should therefore be read as a policy snapshot anchored to the BEACON 2025 reporting frame unless a later date is explicitly stated.
